# Miller–Urey Spark‐Discharge Experiments in the Deuterium World

**DOI:** 10.1002/anie.201610837

**Published:** 2017-05-05

**Authors:** Geoffrey J. T. Cooper, Andrew J. Surman, Jim McIver, Stephanie M. Colón‐Santos, Piotr S. Gromski, Saskia Buchwald, Irene Suárez Marina, Leroy Cronin

**Affiliations:** ^1^ WestCHEM, School of ChemistryUniversity of GlasgowUniversity AvenueGlasgowG12 8QQUK

**Keywords:** analytical chemistry, deuterium, Miller–Urey experiment, origin of life, spark discharge, systems chemistry

## Abstract

We designed and conducted a series of primordial‐soup Miller‐Urey style experiments with deuterated gases and reagents to compare the spark‐discharge products of a “deuterated world” with the standard reaction in the “hydrogenated world”. While the deuteration of the system has little effect on the distribution of amino acid products, significant differences are seen in other regions of the product‐space. Not only do we observe about 120 new species, we also see significant differences in their distribution if the two hydrogen isotope worlds are compared. Several isotopologue matches can be identified in both, but a large proportion of products have no equivalent in the corresponding isotope world with ca. 43 new species in the D world and ca. 39 new species in the H world. This shows that isotopic exchange (the addition of only one neutron) may lead to significant additional complexity in chemical space under otherwise identical reaction conditions.

Understanding life's origin remains one of the greatest challenges to science but it is not possible to make direct observations of what actually happened. Instead, understanding potential sources of key compounds that are found in extant life is a popular approach. With this in mind, attention has focused on determining mechanisms for the formation of monomers for the first “bio” polymers. One important experiment in establishing the field of prebiotic chemistry was the Miller–Urey experiment in the 1950s, where spark discharge through a simulated reducing atmosphere was shown to yield amino acids.^1^ Since that landmark work, several further studies have explored similar systems and brought more advanced analytical techniques to bear on the complex product mixtures produced, as well as arguing if a reducing atmosphere could be realistic for the early Earth.^2^, ^3^, ^4^

As with many such experiments, the Miller–Urey reaction has been typically performed in sealed clean glassware with a small number of pure reagents, whereas life emerged in a far more complex and heterogeneous environment.^5^ However, even this simple system produces a large and diverse range of products, thought to be resulting from a combinatorial explosion of simple uncontrolled reactions, and their study is limited due to their perceived analytical intractability.^6^ Most studies have therefore focused on very specific “prebiotically relevant” products, whereas we hypothesized that a more “systems” approach, studying the emergence of patterns, organization, and other such phenomena, might also give important insights at establishing the pathways by which the chemistry of biology formed. Rather than attempting to find specific chemical species in these mixtures, we are interested in what small changes to the reaction conditions or “environment” can produce the largest overall changes in product distribution of complex chemical networks; that is, what minimal change can we apply to give the greatest degree of complexification?

Herein, we use fully deuterated starting materials in the Miller–Urey spark discharge experiment—a change of one neutron—to investigate whether kinetic isotope effects might have a significant influence on the product distribution (Figure 1). This choice was also inspired by the coincidence that deuterium was discovered by Urey in 1931,^7^ one of his most notable contributions outside of the origin‐of‐life field, and that discovery would not have been possible without the discovery of isotopes by Frederick Soddy while working in Glasgow in 1912.^8^

**Figure 1:**
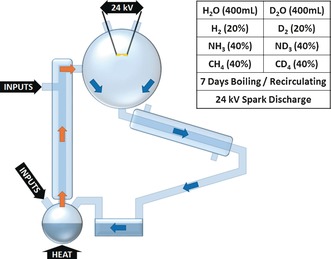
Schematic representation of the spark‐discharge experiment showing the circulation of water vapor (orange arrows) and condensate (blue arrows) as well as the experimental inputs for the deuterated and non‐deuterated experiments.

We found that deuteration of the Miller–Urey system did not result in large changes when subjected to analyses typically performed on such experiments (for amino acids). However, when analysis was performed without bias of product expectations (in this case LC‐MS without selective derivatization), the wider distribution of products was found to be significantly different with differences in the distribution of matched isotopologues and several species that are unique to one or other isotope environment. Deuterated and non‐deuterated Miller–Urey experiments were run for seven days on each of the two sets of apparatus and repeated at least three times. Analysis was performed offline after the experiment was complete. As an initial analysis, and to align our work with that of others in the field, HPLC chromatograms were obtained with derivatization to detect primary amines (such as amino acids) by fluorescence (FLD) detection (Figure 2 a). By comparison with known amino acid standards, we find our mixture to contain glycine, alanine, and β‐alanine, which is consistent with the previous findings of such simple spark‐discharge experiments.^9^, ^10^

**Figure 2:**
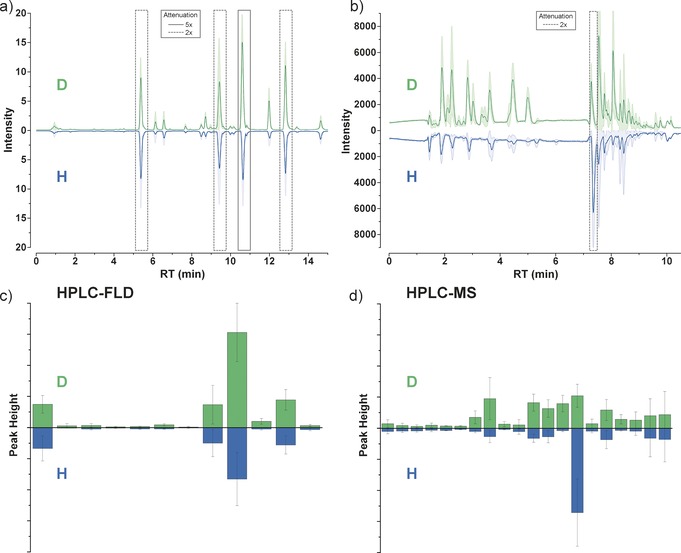
Data analysis of HPLC‐FLD and HPLC‐MS for the H (blue) and D (green) experiments. Plots are mirrored with D on top and H inverted below to allow easy comparison of peak position. a) HPLC‐FLD chromatograms. b) HPLC‐MS base peak chromatograms. In both sets of chromatograms, the most intense peaks are attenuated to allow smaller peaks to also be resolved. c) Bar plot of picked peaks from HPLC‐FLD data. d) Bar plot of picked, matched, peaks from HPLC‐MS data. Raw data for the bar plots can be found in the Supporting Information. Colored areas around the traces of chromatograms and error bars in the bar plots represent the standard deviation over six experimental replicates, each with three analytical repeats.

With the exception of a few subtle changes, the product distribution of primary amines appears to be very similar in both the deuterated and the non‐deuterated experiment (see Figures 2 a and 2 c). Amino acid formation in the Miller–Urey system is known to take place via the Strecker mechanism, the rate‐determining step of that reaction being a deprotonation and subsequent hydrolysis.^11^, ^12^ This might lead to the expectation of a notable difference in product distribution of amino acids between the deuterated and non‐deuterated experiments. However, the observed near‐invariance in the amine product distribution suggests that any differences that might be seen in the kinetics are not great, though following the changes in product distribution during the experiment would be an interesting candidate for a future on‐line analysis.

We note that a large proportion of analytical work in the field of prebiotic chemistry sets out to look for a rather specific product set, and then finds them (or not). Rather than specifically looking for amino acids, or indeed any specific chemical species, we wished to examine a different area of chemical space without the bias of expectation. To approach this, we ran the samples through reverse‐phase HPLC and then directly into a mass spectrometer, without any derivatization reaction. Under these circumstances, very polar materials will not be retained, but most other soluble products can be separated, and we get a data set that represents a rather different portion of the product‐space to the HPLC‐FLD data described above. Comparison of the base‐peak chromatograms (BPCs) produced reveals many similarities but also some gross differences in the peak distributions and intensities between the H and D experiments (see Figure 2 b).

Typically, intensities associated with different species in HPLC‐MS data sets would be compared, to assess difference between two systems. However, this was complicated by the fact that that isotopologues appear as different products in MS analysis (different *m*/*z*; same retention time, RT). To overcome this difficulty, we used a peak‐picking algorithm to obtain a list of individual unique features in each HPLC‐MS analysis. A list of these coordinates (*m*/*z*, RT) for features picked in the H experiments was then cross‐referenced against the lists resulting from D experiment data, searching for cases where an equivalent peak could be found (*m*/*z* from H experiment plus an integer number of deuterium substitutions; same RT). Comparison of this list of “compounds” with raw data revealed that the majority of large peaks in the BPC were accounted for by this list of products.

Plotting the peak heights of these species we can clearly see that there is a significant difference between the products of the deuterated system compared to the non‐deuterated (see Figure 2 d). It can be observed that the products from the H experiments are overwhelmingly dominated by one species, whereas the products of the D experiments are richer in a wide range of products. That this molecular diversity can be provoked by such a simple isotopic substitution is surprising.

A prominent feature of “systems” science is the need to use statistical analysis to simplify large complex datasets which cannot be readily understood “by eye”. Multivariate statistical tools such as principle component analysis (PCA) and discriminant analysis (DA) are powerful methods for providing an overview of complex data.^13^, ^14^ As perhaps the archetypal “systems chemistry” experiment, it is surprising that such an approach has not been applied to product distributions in Miller–Urey experiments.^15^, ^16^ Applying simple PCA to our data, reveals that a systematic distinction between the product sets of H and D can be observed (see Figure 3). This is much more of a true “systems” approach as we now consider the ensemble rather than individual components. This may perhaps be attributed to the greater variance in the data resulting from HPLC‐FLD, and the greater information‐richness of HPLC‐MS (data as intensity vs. RT and *m*/*z*, rather than just intensity vs. RT). In addition, principal component‐discriminant function analysis (PC‐DFA) was performed on GC‐MS data, which provides a “fingerprint” of the mixture without any peak‐picking bias. Here too, a clear and systematic difference is observed (see Supporting Information).

**Figure 3:**
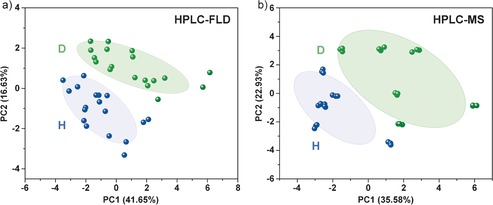
PCA of picked peaks from the H (blue) and D (green) experiments a) HPLC‐FLD and b) HPLC‐MS analysis. Confidence ellipses represent one standard deviation and loadings can be found in the Supporting Information.

We find that there is considerable variance in the data, which has perhaps not been addressed previously. Indeed, we are unaware of any work in the field where data from multiple experimental replicates, or experiments from different apparatus, are compared in this way. Inspecting our data, we can observe variance in the products of experimental replicates, although broad trends do remain. Furthermore, we can even see a difference in the data depending on which set of apparatus was used, although they were nominally identical. Just as we note above that a strikingly great product diversity is observed in the D experiments, distribution of material between these possible products appears to be more subject to small fluctuations.

In addition to variance between experiments and apparatus, there are clear trends in reproducibility between the two analytical techniques employed. The difference between repeat analyses of the same product mixtures in HPLC‐FLD data is considerably greater than that observed in HPLC‐MS. We attribute the greater robustness of HPLC‐MS analysis to its providing and extra dimension of for resolution (intensity vs. *m*/*z* and RT), which is particular important to resolve species in complex mixtures with great dynamic range. While variance of HPLC‐FLD data might also be attributed to some irreproducibility of derivatization reactions in complex mixtures, using this method^17^ good reproducibility has been observed for simple mixtures of amino acids, and the contribution can be discounted. In addition, the small difference in RT observed for H vs. D isotopologues by others^18^ is expected to be considerably smaller than the deviation allowed by our matching algorithms. We note very little change in retention time between matched species in the H and D experiments.

Analysis of some 120 picked peaks (RT‐*m*/*z* coordinates) from the HPLC‐MS data reveals approximately 40 that can be assigned a match in the corresponding H or D data, and the matched peaks make up the majority of large peaks. While the identity of the materials within the mixture is unimportant for the main argument of this work, tentative formula fitting using mass and valance rules^19^ shows that the product mixture is consistent with an ensemble of organic species between C_2_ and C_13_ in the mass range 100–350 Da (see Supporting Information). With the matched species, we were also able to fit matching formulae with plausible levels of deuteration. None of the suggested formulae is fully deuterated, as is expected owing to exchange with non‐deuterated solvents during analysis. The remaining majority of picked peaks (ca. 80) are found only in the “hydrogenated” or “deuterated” worlds and it is interesting to note that the addition of one neutron produces such a difference under chemically identical reaction conditions.

In conclusion, we have shown that there are significant differences in the overall product distribution of the Miller–Urey system when comparing the non‐deuterated and deuterated experiments, and that many species are unique to one or other isotope environment. This far more of a “systems” approach, and it is of particular interest that these largest differences between D and H are seen in the non‐polar products, which we are currently investigating further. Analysis of these systems is usually steered towards amino acid detection by use of derivatizing agents to reduce analytical complexity. While the products from the H experiments are overwhelmingly dominated by one species, the products of the D experiments are richer in a wide range of products, and it is striking that this molecular diversity can be provoked by such a simple isotopic substitution. These clear differences in the product distributions were not only found to arise between different isotopes, but also between experimental set‐ups, as well as within a given experiment. These results are exciting as they show how random fluctuations play a major part in determining product distribution. Further work aims to explore this in great detail aiming to couple the fluctuations to catalytic processes to allow amplification, as well as further identification of product classes and mapping compositional space to functionality.

## Conflict of interest

The authors declare no conflict of interest.

## Supporting information

As a service to our authors and readers, this journal provides supporting information supplied by the authors. Such materials are peer reviewed and may be re‐organized for online delivery, but are not copy‐edited or typeset. Technical support issues arising from supporting information (other than missing files) should be addressed to the authors.

Supplementary
