# Supplementary material for: Miller–Urey Spark‐Discharge Experiments in the Deuterium World
Source: Angew Chem Int Ed Engl. 2017 May 5;56(28):8079–82. doi: 10.1002/anie.201610837 (PMC5499724; doi:10.1002/anie.201610837)
Supplement: Supplementary file 1 — Supplementary [file ANIE-56-8079-s001.pdf]

Angewandte Chemie
Eine Zeitschrift der Gesellschaft Deutscher Chemiker

Supporting Information

Miller–Urey Spark-Discharge Experiments in the Deuterium World

*Geoffrey J. T. Cooper, Andrew J. Surman, Jim McIver, Stephanie M. Colón-Santos, Piotr S. Gromski, Saskia Buchwald, Irene Suárez Marina, and Leroy Cronin**

anie_201610837_sm_miscellaneous_information.pdf

Table of Contents

Experimental methods and apparatus schematic. Figure S1. 2

HPLC-FLD plots with picked peaks and blanks. Figures S2 & S3. 4

HPLC-MS polts with picked peaks and blanks. Figurees S4 & S5. 5

HPLC-FLD plot with standard amino acids. Figure S6. 6

GCMS TIC plots for H and D. Figures S7 & S8. 8

PCA scores and loadings plots. Figures S9 & S10. 9

PCA scores and PC-DFA histogram for GC-MS raw data. Figures S11 & S12 10

PCA scores and PC-DFA histogram for HPLC-UV raw data. Figures S13 & S14 11

EICs of picked peaks used in PCA. Figures S15 – S21. 12

Picked peaks from HPLC-FLD data. Table S1. 14

Picked peak deuterations for HPLC-MS data. Table S2. 15

Picked peaks from HPLC-MS data. Tables S3 & S4. 16

Tentative assignment of formulae to picked peaks. Tables S5, S6, S7 & S8. 18

Experimental Methods

Reagents and Gases: Chemical reagents were obtained from Sigma-Aldrich and were used without further purification. HPLC grade water was used and deuterium oxide was supplied by Goss Scientific. Gas mixtures were supplied pre-mixed by the British Oxygen Company (BOC) and CK Special Gases Ltd.

Experimental Procedure: Two sets of spark discharge apparatus were built (schematic in Figure S1). A typical experiment was carried out as follows: After careful cleaning and drying of the glassware, 400 mL of HPLC grade water (or deuterium oxide) was added and the system sealed. The whole rig was pumped down three times to de-gas the water and finally after the third evacuation, the system was pressurized to 1 atm with gas mixture (40% methane, 40% ammonia and 20% hydrogen, or their deuterated equivalents). Heating was applied to the main flask and once boiling and recirculation was established, the 24 kV spark discharge was turned on with a 10 sec alternating ON / OFF duty-cycle. Experiments were run for seven days, during which time the solution in the flask became deep brown in colour. After the spark discharge / heating was turned off and the system had cooled, the contents of the flask was collected and the rig cleaned, during which any solid residues were collected. The experiment was repeated three times on each set of apparatus for both the non-deuterated and deuterated system. In the data presented, X1 and X2 refer to the two experimental setups (nominally identical and merely used to allow faster acquisition of experimental replicates), while experimental replicates are denoted as H1, H2 & H3 and D1, D2 & D3 for the non-deuterated and deuterated experiments respectively.

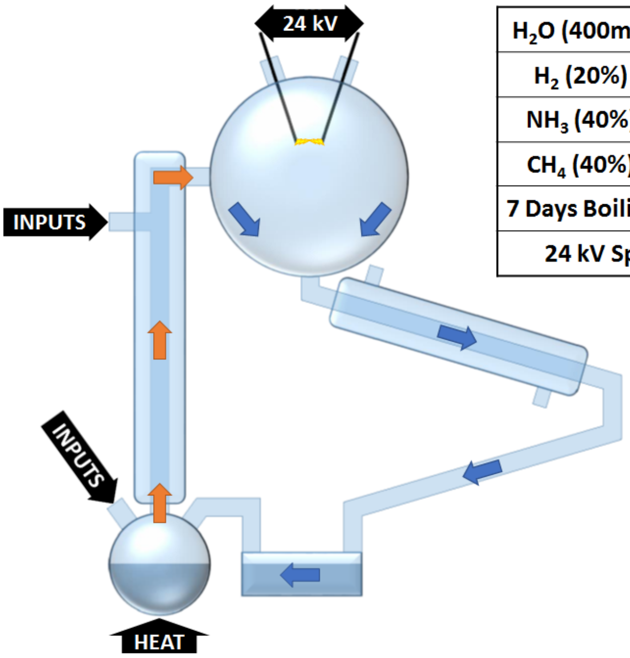

H_2O (400mL)	D_2O (400mL)
H_2 (20%)	D_2 (20%)
NH_3 (40%)	ND_3 (40%)
CH_4 (40%)	CD_4 (40%)
7 Days Boiling / Recirculating	
24 kV Spark Discharge	

Figure S1. Schematic of the spark-discharge experiment showing the circulation of water vapour (orange arrows) and condensate (blue arrows) as well as the experimental inputs.

Analytical Methods: Samples of 400mL volume were collected after 7 days for each experiment and placed in 1 litre Duran bottles (PYREX), which were stored at room temperature before analysis.

HPLC-FLD: HPLC-FLD analyses were performed using an Agilent 1200 HPLC system, following a standard protocol method for the analysis of amino acids,[1] involving automated derivitisation of amine groups with o-phthalaldehyde (OPA)/mercaptopropionic acid (MPA) and 9-fluorenylmethyloxycarbonyl chloride (FmocCl) to allow retention of the products on a reverse phase column (Agilent Poroshell 120 HPH C18, 3.0 x 100 mm, 2.7 µm) and detection using a fluorescence detector (excitation at 340 nm, emission detected at 450 nm). The instrument was controlled and data acquired using Agilent OpenLab software. Three identical analyses were recorded for each experimentally-produced sample; in addition, a series of standards of products identified in previous spark discharge experiments were analysed for comparison (and as a means to confirm the absence of significant retention time (*rt*) drift; see SI for

details). The same software was used to detect and integrate all significant peaks, and extract corresponding intensities (peak height) in all runs (+/- 2% retention time 'window'; also checked manually).

HPLC-MS: HPLC-MS analyses were performed with Thermo Ultimate 3000 UPLC system fitted with an Agilent Poroshell 120 EC-C18 (4.6 × 150 mm, 2.7 µm) column. Samples were injected in 5 µl aliquots and eluted with a linear gradient mixture of solvents A (water w/0.1% v/v formic acid) and B (100% acetonitrile w/0.1% v/v formic acid) over 26 min as follows: 0 min, 100% A; 4min, 100% A; 16 min, 70& A & 100% B; 19 min, 100% B; 23 min, 100% B; 23min, 100% A. The column was maintained at 30 °C. The MS apparatus was a Bruker MaXis Impact instrument, calibrated for the 50–1,200 Da range using sodium formate solution. The eluent stream was introduced directly into the source (no splitting) following the diode array detector, at a dry gas temperature of 200 °C. The ion polarity for all MS scans recorded was positive, with the voltage of the capillary tip set at 4,800 V, end plate offset at −500 V, funnel 1 RF at 400 Vpp and funnel 2 RF at 400 Vpp, hexapole RF at 100 Vpp, ion energy 5.0 eV, collision energy at 5 eV, collision cell RF at 200 Vpp, transfer time at 100.0 µs and the pre-pulse storage time at 1.0 µs. The instruments were controlled and data acquired using Bruker Hystar & Compass software, along with Thermo DCMS Link. Bruker Compass was then used to 'dissect' data sets, producing a list of peaks observed. The parameters used in the internal 'DataAnalysis Dissect' were signal/noise threshold: 3, max. overlapping compounds: 5, spectrum type: auto, and cut-off intensity: 0.1%. Custom scripts in R[2] were used to match peaks corresponding to isotopologues observed in both 'H' and 'D' experiments by searching for features with matching retention times (+/- 20 s) and *m/z* corresponding to up to 20 H/D exchanges. Intensities (peak height) corresponding to each 'picked peak' were then extracted from all the raw data files (not just those in which the feature was observed as follows: ion chromatograms were extracted for each mass (using the xcms[3] library) from the data files (following conversion of MS files to mzML format using Proteowizard)[4], and a maximum intensity corresponding to the coordinates of each picked peak (retention time +/- 15 s; *m/z* +/- 0.01 Da).

GC-MS: Sample preparation: 10 mL of each sample was transferred into falcon tubes and centrifuged for 10 minutes at 4,000 rpm. 1mL of the supernatant is transferred to a spin filter (filter cut-off 0.2µm) and centrifuged for 10 minutes at 4,000 rpm. This was done twice and the filtered fraction transferred into sample vials (total volume per sample: approx. 1.5 mL to 1.8 mL). Sample vials are then frozen (by placing them at a -20 °C for an hour) and lyophilized. *Derivatization reaction:* 45µL of MTBSTFA and 5 µL of Acetonitrile (HPLC grade) are added to the dried sample (in the sample vial), then placed in a ultra-sonic bath with a frequency of 37 Hz at a temperature range of 50 C° to 60 °C for 60 minutes. *After derivatization (and prior to GC-MS analysis)* samples are diluted 1:10 in Acetonitrile (HPLC grade) and filtered using a syringe filter with a 0.22 µm cut-off. *GC method:* The analyses were performed using an Agilent 7890 GC / 5975 MSD. 2 µL per sample was injected into the GC in split-mode 1:20. The injector temperature was set at 250 °C and the detector at 230 °C. Helium was used as the carrier gas at a constant flow of 1.0 mL/min. An HP-5MS capillary column (95% dimethylpolysiloxane, 5% diphenyl; 30m x 0.25m x 0.25 mm) from Agilent J&W was programmed at 75 °C, hold temperature for 3 minutes, then 3 °C/per minute to 140 °C, hold temperature for 3 minutes, then 3 °C/per minute to 200 °C, hold temperature for 1 minute, then 5 °C/per minute to 230 °C.

Principal component analysis (PCA): Both of the resulting data sets (HPLC-FLD and HPLC-MS, obtained and prepared as outlined above) were subjected to simple PCA with scaling of the raw data (using the FactoMineR[5] library in R). In the manuscript figures confidence ellipses correspond to a 68% confidence limit (one standard deviation) and were calculated in R using the 'dataEllipse' function from the car package.[6] For the GC-MS data, multiplative signal correction was applied before the PCA analysis.[7]

Principal component-discriminant function analysis (PC-DFA): DFA separates two or more groups by looking for a linear combination of variables in the subspace that maximises the between-group distances and simultaneously reducing within-group distances.[8] As this study was a binary classification (H vs. D) only a single latent variable can be calculated and therefore we have displayed this as a histogram. The

number of components used for DFA was estimated through visual assessment of a generalized scree plot, which demonstrates the variance associated with each component in descending order versus the number of components. In this case, three components, representing 78.98% of variance, where selected. R packages “stats” and “MASS”[9] were used herein.

Formula assignment: Formula assignment was done using a script in R and limited to the elements carbon, hydrogen (and deuterium), nitrogen and oxygen and the following elemental ratios: H/C min=0.1 max=6, N/C min=0 max=4 & O/C min=0 max=3.[10] Suitable formulae with the best ppm error were picked from the possible outputs. All formulae are fitted to the m/z value and assumed to represent $[M+H]^+$ ions. While other adducts are possible under ESI conditions, the assumption that species would be $[M+H]^+$ allowed tentative assignment of possible formulae. Since the identification of species is outside the remit of this work, more in-depth analysis to determine formulae was not persued.

References

[1] W. Long, *Agilent Technologies, Inc.*, Application Note. 5991-5571EN. **2015**

[2] R Core Team; R: A language and environment for statistical computing. R Foundation for Statistical Computing, Vienna, Austria. **2015** URL https://www.R-project.org/.

[3] C. A. Smith, E. J. Want, G. O'Maille, R. Abagyan, G. Siuzdak, *Anal. Chem.* **2006**, *78*, 779–787.

[4] M. C. Chambers, *Nature Biotech.* **2012**, *30*, 918–920.

[5] S. Lê, J. Josse, F. Husson, *J. Stat. Softw.* **2008**, *25(1),* 1-18.

[6] J. Fox, S. Weisberg, An {R} Companion to Applied Regression, Second Edition. Thousand Oaks CA: Sage. **2011** URL: http://socserv.socsci.mcmaster.ca/jfox/Books/Companion

[7] H. Martens, T. Næs, *Multivariate calibration.* **1989** Chichester: Wiley.

[8] P. S. Gromski, Y. Xu, K. A. Hollywood *et al. Metabolomics* **2015**, *11*, 684.

[9] W. N. Venables, B.D. Ripley, *Modern Applied Statistics with S. 4th ed*. **2002**, New York, NY, USA: Springer.

[10] T. Kind, O. Fiehn, BMC Bioinformatics **2007**, *8:105*.

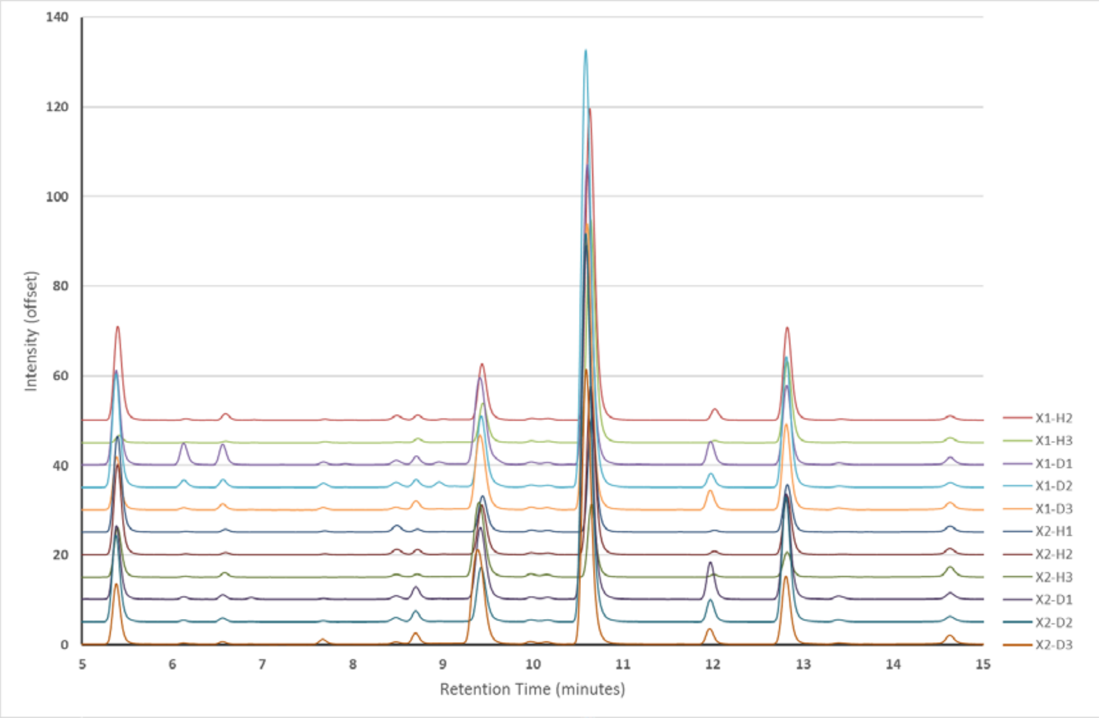

Figure S2. HPLC-FLD plots of all the experimental runs.

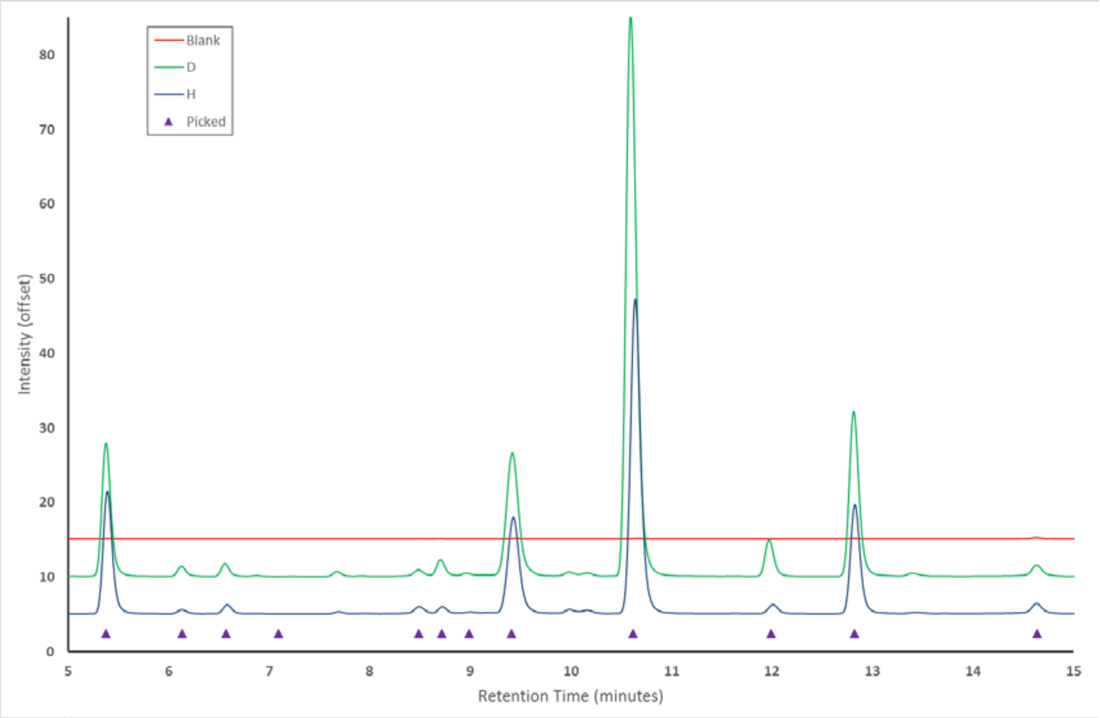

Figure S3. HPLC-FLD plots averaged for deuterated vs. non-deuterated, compared to a blank run. Purple triangles show the peaks that were picked in the data analysis.

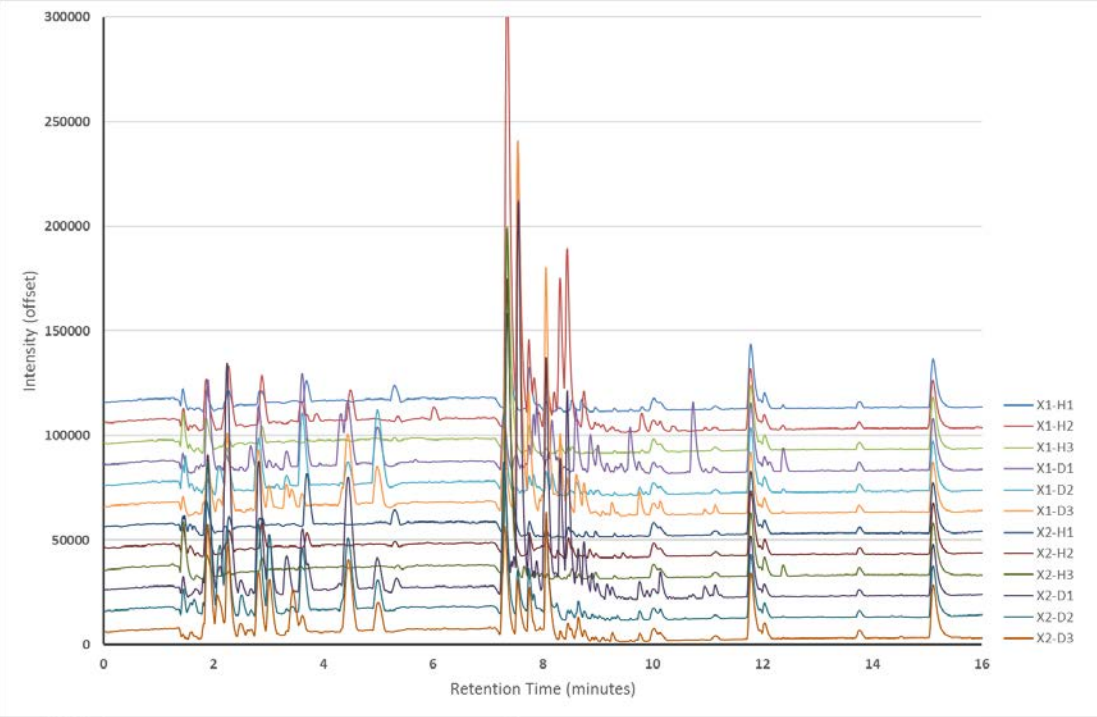

Figure S4. HPLC-MS BPC plots of all the experimental runs.

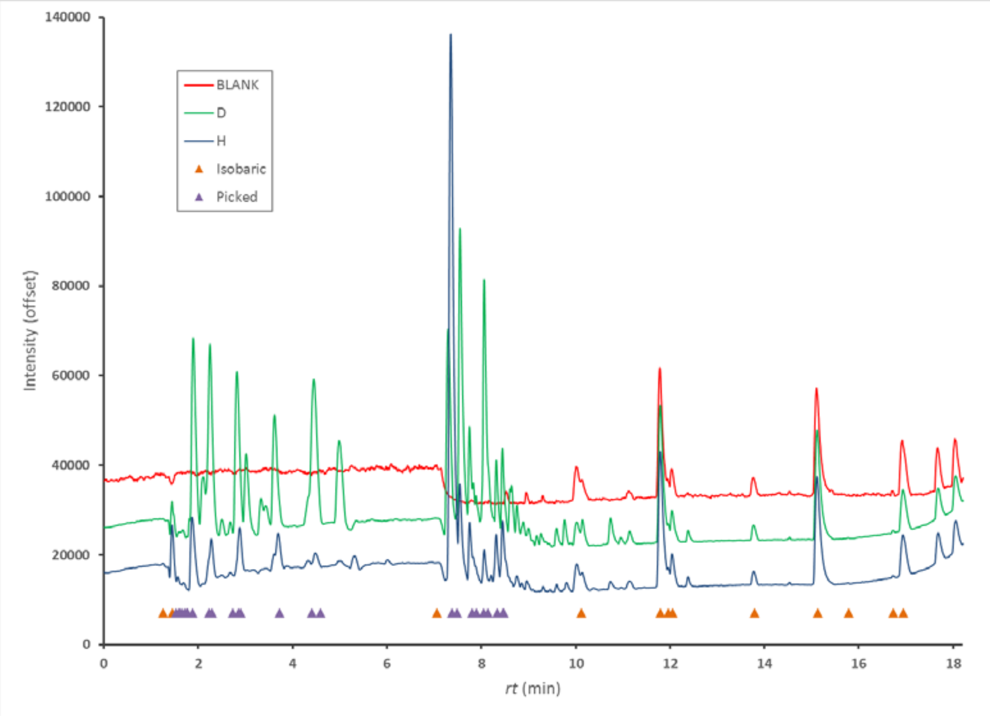

Figure S5. HPLC-MS BPC plots averaged for deuterated vs. non-deuterated, compared to a blank run. Purple triangles show the H peaks that were picked in the data analysis and matched to D isotopologues. Orange triangles mark peaks that were identified in both H and D but were found to have the same *m/z* and therefore were not considered to originate from the MU system.

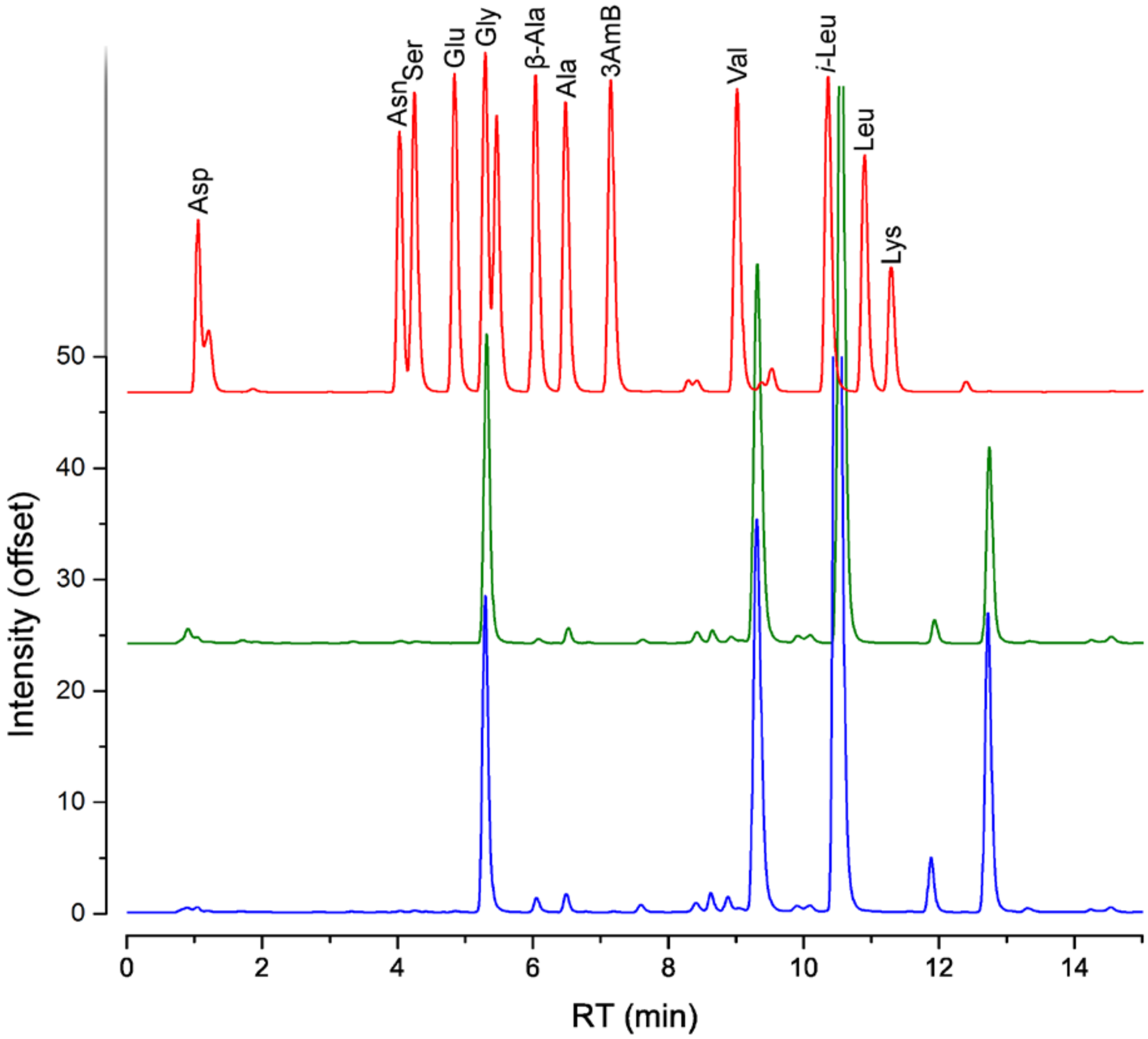

Figure S6. HPLC-FLD plots of amino acid standards (red, top), a representative deuterated run (green, middle) and a representative non-deuterated run (blue, bottom), showing positive identification of glycine and tentative identification of alanine and β-alanine. Standards of aspartic acid, asparagine, serine, glutamine, glycine, β-alanine, alanine, 3-amino butyric acid, valine, iso-leucine, leucine and lysine were made up at 2.5mM in HPLC grade water.

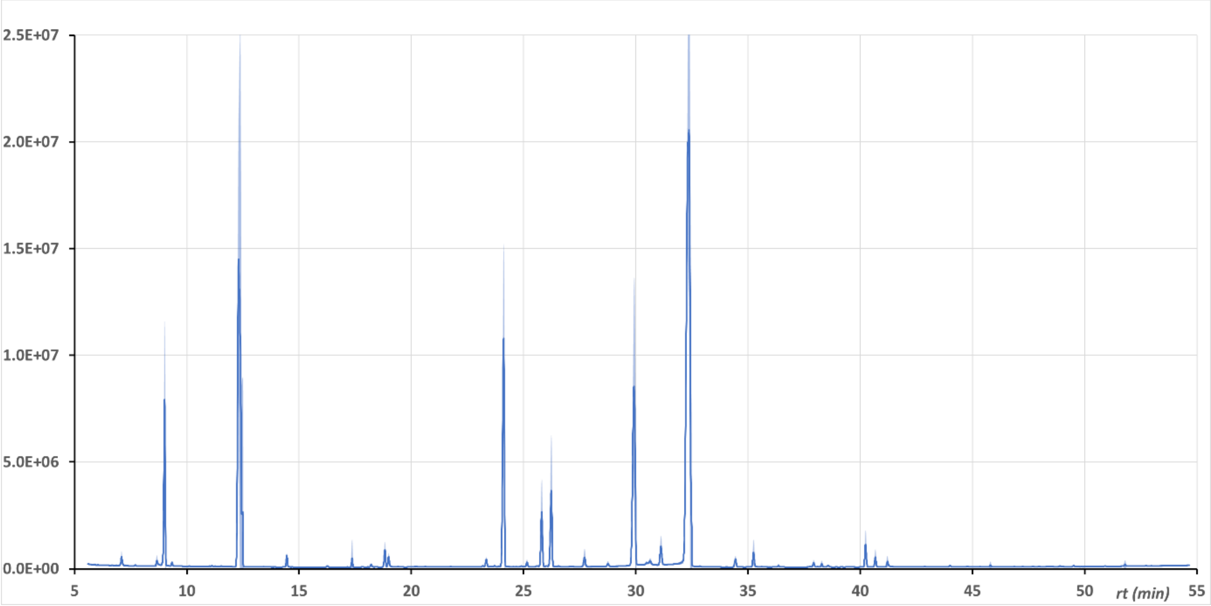

Figure S7. GC-MS Total Ion Chromatogram for H experiments (MTBSTFA derivatisation). Coloured areas around the traces represent the standard deviation over six experimental replicates and three analytical repeats.

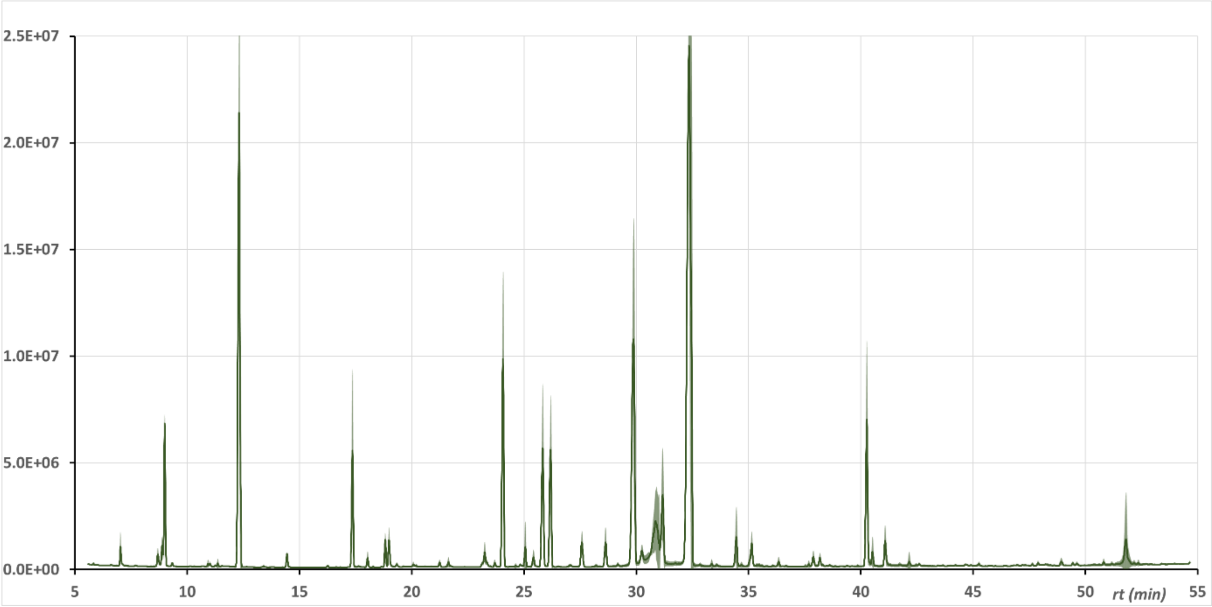

Figure S8. GC-MS Total Ion Chromatogram for D experiments (MTBSTFA derivatisation). Coloured areas around the traces represent the standard deviation over six experimental replicates and three analytical repeats.

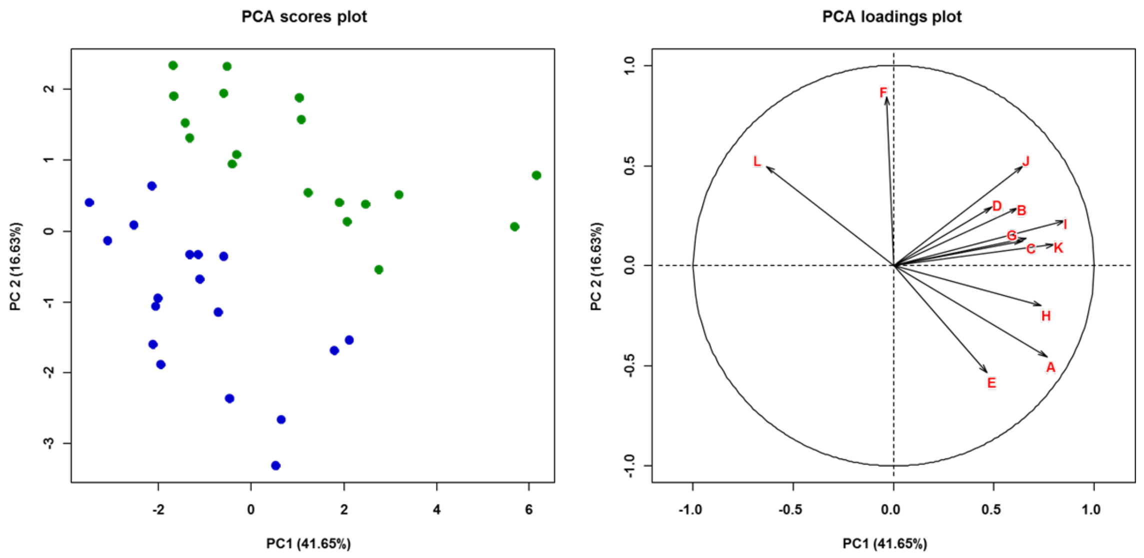

Figure S9. Simple PCA of picked peaks from HPLC-FLD data for H (blue) and D (green) with scores plot on the left and loadings plot on the right. Peak numbers correspond to the headers in table S1.

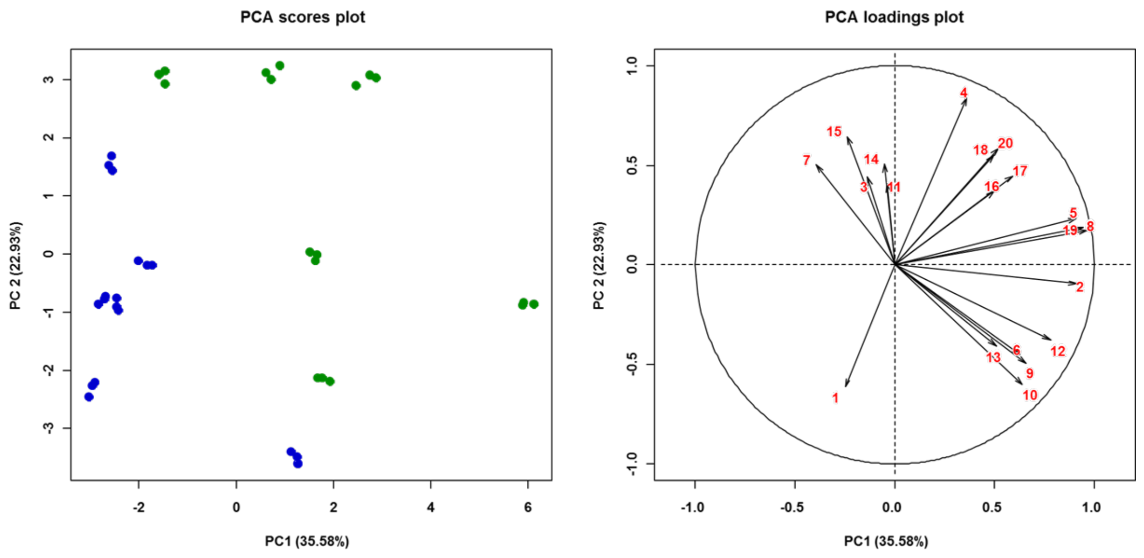

Figure S10. Simple PCA of picked peaks from HPLC-MS data for H (blue) and D (green) with scores plot on the left and loadings plot on the right. Peak numbers correspond to the headers in tables S3 and S4.

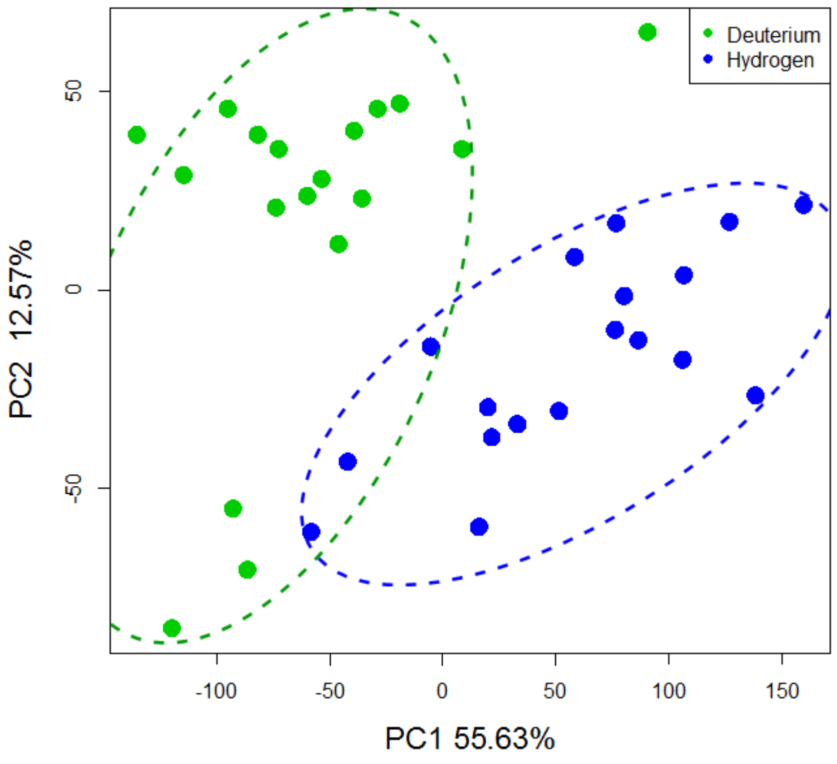

Figure S11. Simple PCA scores plot of GC-MS raw data (no peak picking / MTBSTFA derivatisation) for H (blue) and D (green). Dotted ellipses are drawn as a guide to the eye and are not calculated confidence ellipses.

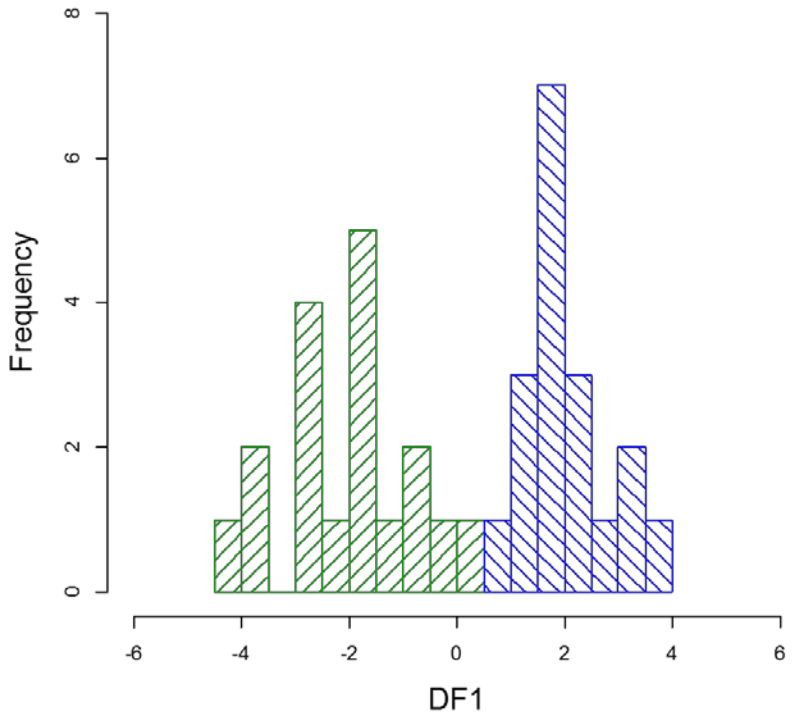

Figure S12. PC-DFA histogram plot of separation in GC-MS raw data (no peak picking / MTBSTFA derivatisation) between Deuterium (green) and Hydrogen (blue) experiments.

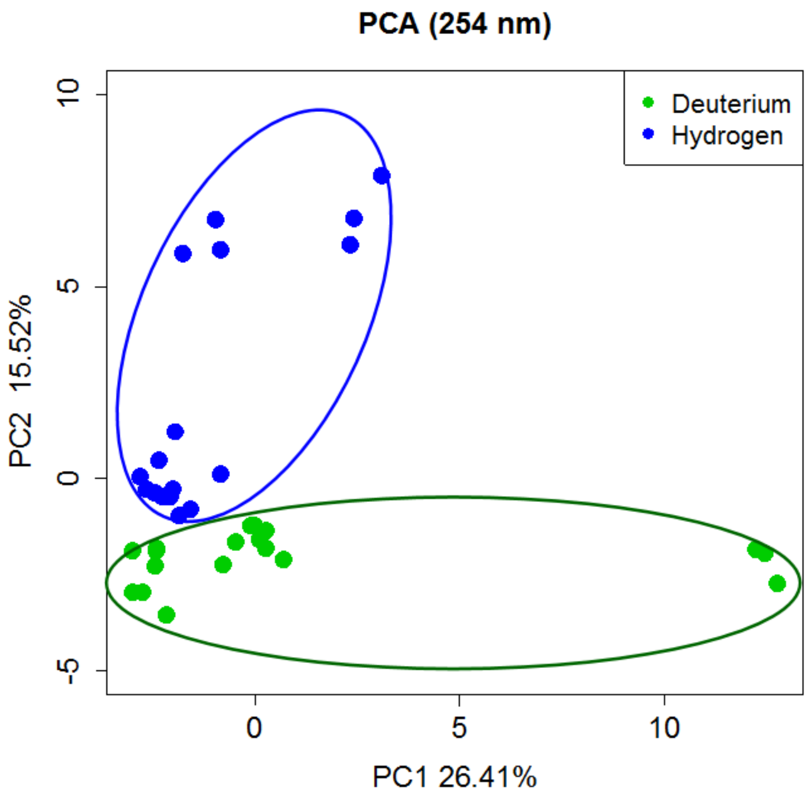

Figure S13. Simple PCA scores plot of HPLC-UV raw data (no peak picking / OPA-NAC derivatisation) for H (blue) and D (green).

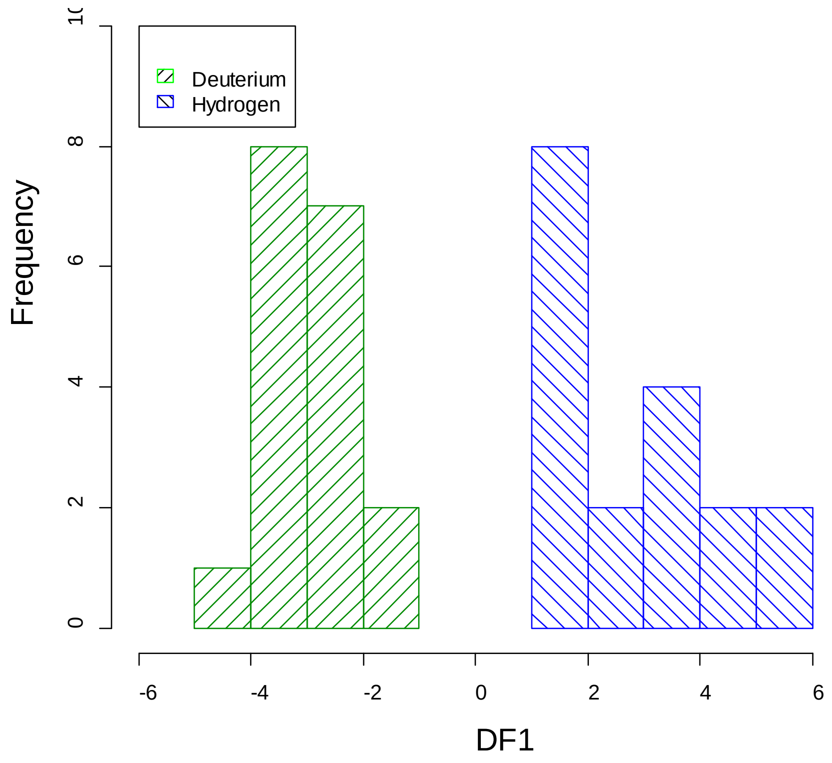

Figure S14. PC-DFA histogram plot of separation in HPLC-UV raw data (no peak picking / OPA-NAC derivatisation) between Deuterium (green) and Hydrogen (blue) experiments.

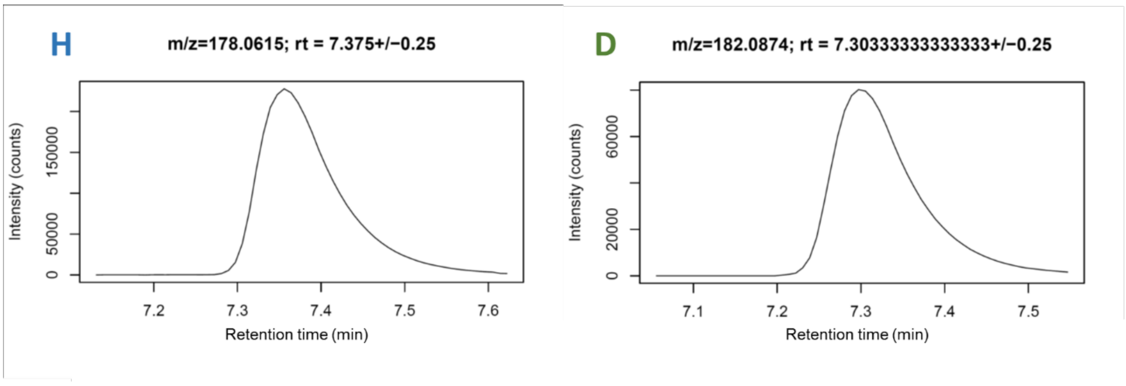

Figure S15. EICs for key matched features in H (m/z 178.0615) and D (m/z 182.0874).

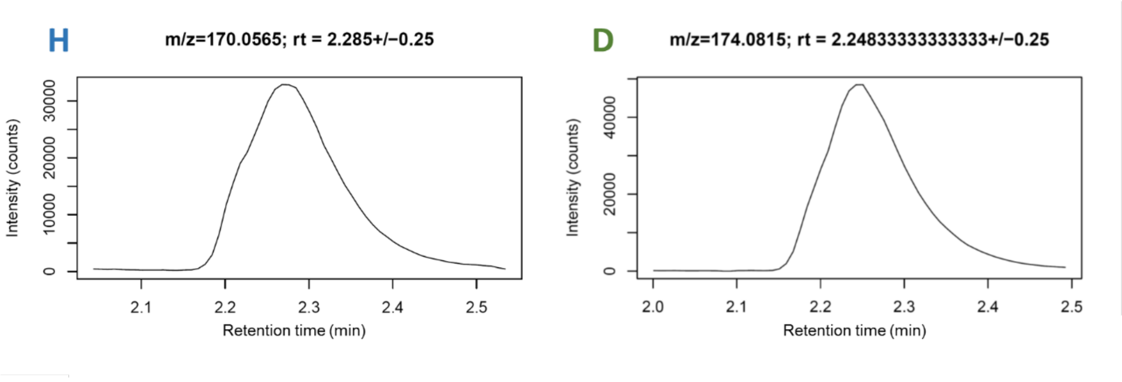

Figure S16. EICs for key matched features in H (m/z 170.0565) and D (m/z 174.0815).

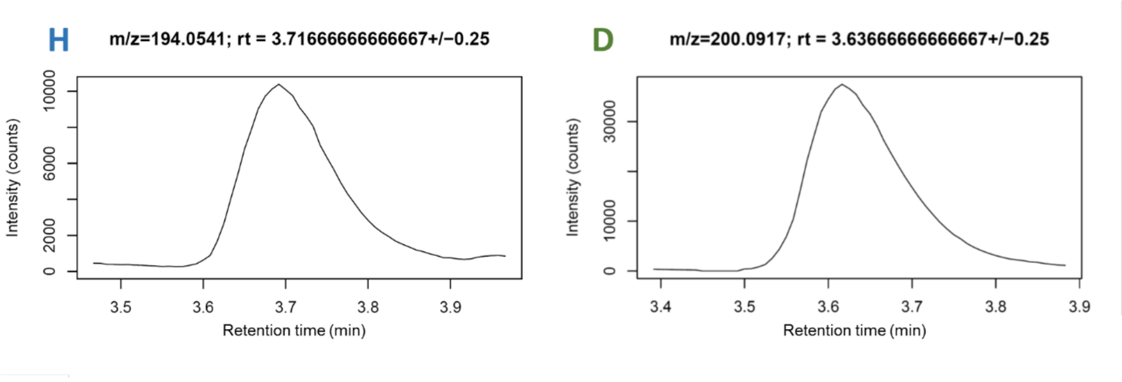

Figure S17. EICs for key matched features in H (m/z 194.0541) and D (m/z 200.0917).

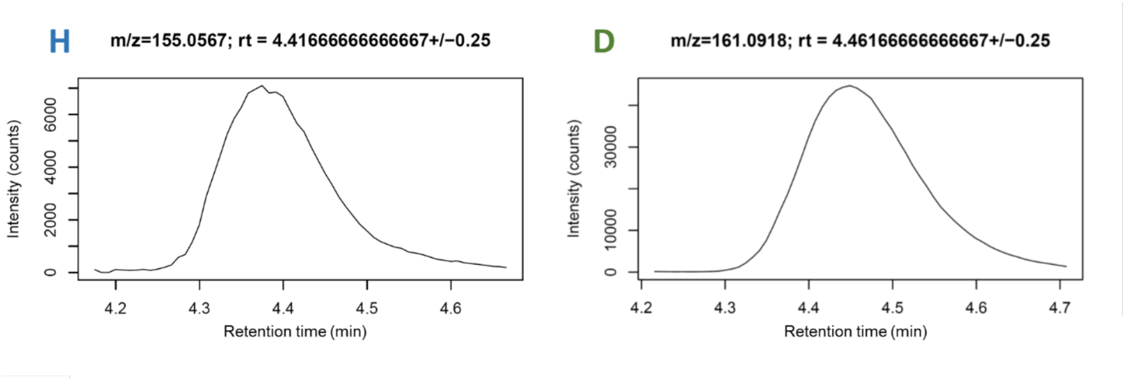

Figure S18. EICs for key matched features in H (m/z 155.0567) and D (m/z 161.0918).

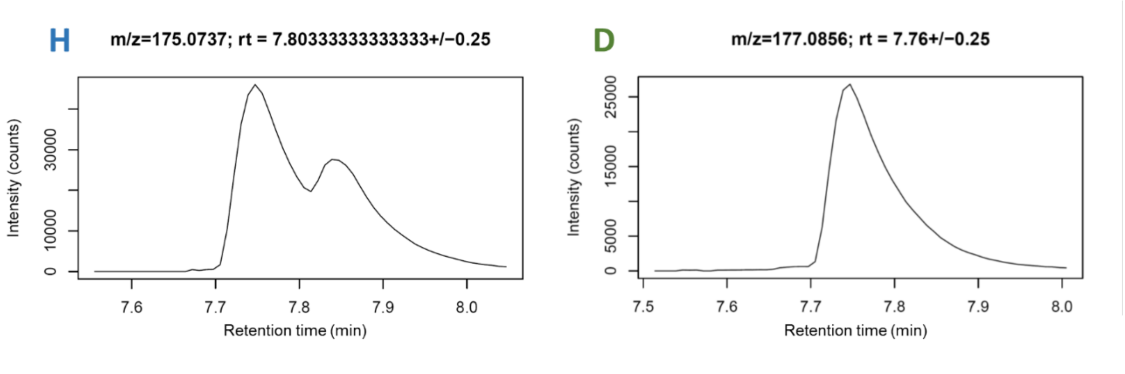

Figure S19. EICs for key matched features in H (m/z 175.0737) and D (m/z 177.0856).

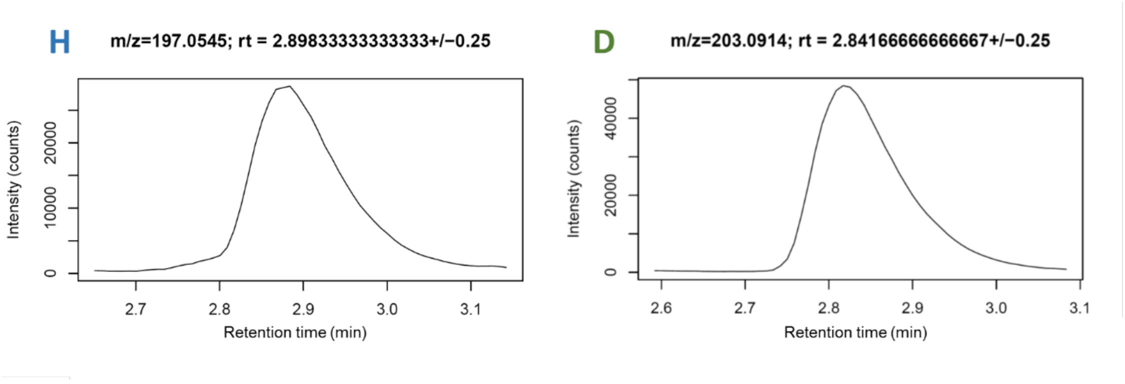

Figure S20. EICs for key matched features in H (m/z 197.0545) and D (m/z 203.0914).

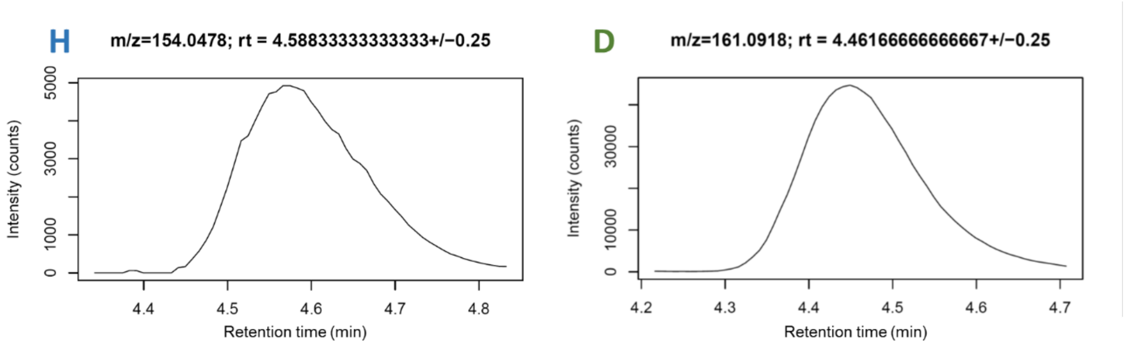

Figure S21. EICs for key matched features in H (m/z 154.0478) and D (m/z 161.0918).

Table S1. Picked peaks from the HPLC-FLD data. Retention times are in minutes and peak heights are an arbitrary unit form the fluorescence detector.

Peak ID	*A*	*B*	*C*	*D*	*E*	*F*	*G*	*H*	*I*	*J*	*K*	*L*
rt (min)	5.38	6.14	6.57	7.09	8.49	8.72	8.99	9.41	10.62	12.00	12.82	14.63
MU_X1_H1_A	27.692	0.525	2.730	0.563	1.138	0.702	0.693	30.847	63.028	1.986	18.571	0.513
MU_X1_H2_A	27.895	0.450	1.882	0.310	1.419	0.783	0.402	22.161	86.461	3.238	34.290	0.853
MU_X1_H3_A	2.192	0.152	0.465	0.366	0.150	0.498	0.117	17.892	68.348	0.641	24.505	0.634
MU_X2_H1_A	34.143	0.299	0.970	0.497	1.986	0.316	0.218	17.384	39.198	0.675	15.966	0.992
MU_X2_H2_A	31.327	0.321	0.696	0.323	1.430	0.636	0.259	23.927	56.507	1.141	17.667	0.555
MU_X2_H3_A	21.631	0.440	1.653	0.029	0.811	0.443	0.111	41.610	29.409	1.014	7.924	0.608
MU_X1_D1_A	30.498	6.851	6.136	0.960	1.354	1.394	0.880	45.443	91.818	7.274	25.792	0.568
MU_X1_D2_A	33.402	2.165	2.215	1.233	1.439	1.514	2.025	29.809	120.373	3.940	40.681	0.532
MU_X1_D3_A	17.449	0.708	1.774	0.756	0.732	1.178	0.233	37.713	90.225	6.177	27.752	0.636
MU_X2_D1_A	23.739	0.859	1.434	0.361	0.913	1.457	0.232	36.384	110.327	11.665	32.118	0.475
MU_X2_D2_A	27.641	0.491	1.182	0.352	1.143	1.317	0.184	26.289	115.836	6.665	36.635	0.471
MU_X2_D3_A	20.546	0.434	0.854	1.685	0.613	1.564	0.225	53.914	89.307	5.085	21.286	0.495
MU_X1_H1_B	17.778	0.331	1.959	0.376	1.051	1.137	0.221	6.732	39.318	1.329	13.838	2.827
MU_X1_H2_B	19.415	0.314	1.295	0.203	0.688	0.785	0.136	8.649	64.023	2.173	10.820	0.655
MU_X1_H3_B	1.298	0.580	0.312	0.222	0.135	1.259	0.827	4.107	40.241	0.397	16.060	1.657
MU_X2_H1_B	16.821	0.144	0.573	0.236	1.424	1.010	0.116	3.303	18.379	0.354	8.618	1.945
MU_X2_H2_B	16.220	0.167	0.434	0.168	1.187	1.517	0.137	5.009	28.729	0.643	11.541	1.981
MU_X2_H3_B	6.055	0.132	0.709	0.641	0.575	0.885	0.134	6.070	9.168	0.384	4.771	3.746
MU_X1_D1_B	17.769	3.989	3.979	0.572	0.979	2.467	0.621	9.571	54.542	4.388	14.358	2.506
MU_X1_D2_B	24.123	1.525	1.629	0.894	1.072	1.756	1.005	11.756	90.278	2.846	23.978	1.399
MU_X1_D3_B	9.693	0.396	1.108	0.438	0.551	2.518	0.217	8.601	51.613	3.564	15.522	2.429
MU_X2_D1_B	13.722	0.495	0.955	0.217	0.791	3.759	0.211	7.603	65.156	6.903	20.672	2.247
MU_X2_D2_B	16.842	0.298	0.800	0.219	0.996	3.098	0.156	5.857	73.729	4.237	24.726	1.783
MU_X2_D3_B	10.825	0.234	0.523	0.932	0.504	3.293	0.193	10.130	49.443	2.853	12.781	3.032
MU_X1_H1_C	18.563	0.336	1.951	0.379	0.913	0.955	0.241	8.134	41.785	1.341	11.895	2.059
MU_X1_H2_C	18.845	0.293	1.360	0.217	1.035	1.836	0.224	7.265	58.713	2.147	17.404	1.486
MU_X1_H3_C	1.388	0.580	0.307	0.231	0.137	1.012	0.106	4.631	42.410	0.402	14.143	1.131
MU_X2_H1_C	17.399	0.149	0.565	0.242	1.265	0.821	0.116	3.979	19.868	0.362	7.579	1.258
MU_X2_H2_C	15.654	0.163	0.415	0.163	1.090	1.378	0.132	4.777	28.308	0.623	10.376	1.708
MU_X2_H3_C	6.738	0.145	0.700	0.641	0.517	0.749	0.125	7.228	10.309	0.400	4.182	2.586
MU_X1_D1_C	17.925	4.017	3.898	0.578	0.949	2.116	0.616	9.792	55.462	4.374	13.566	2.268
MU_X1_D2_C	22.058	1.401	1.509	0.838	1.117	2.062	0.738	7.499	83.102	2.625	23.118	1.538
MU_X1_D3_C	9.735	0.379	1.089	0.444	0.520	2.505	0.207	7.840	52.642	3.562	14.322	1.881
MU_X2_D1_C	13.293	0.476	0.898	0.209	0.748	3.469	0.200	6.939	62.901	6.560	18.958	1.817
MU_X2_D2_C	16.601	0.274	0.773	0.215	0.931	2.986	0.158	5.667	73.175	4.142	22.274	1.548
MU_X2_D3_C	10.709	0.232	0.504	0.911	0.484	2.814	0.197	9.868	48.960	2.779	11.860	2.730

Table S2. Picked peaks from the non-deuterated HPLC-MS data where equivalent deuterated isotopologue peaks have been identified, along with the number of D substitutions. Retention times are in minutes. Isobaric peaks (those where there is no D substitution found) from the blank runs as well as all experiments are shown in grey.

rt	*Max m/z*	*No. D*
1.53	154.060	4
1.59	102.056	7
1.60	148.061	7
1.66	213.050	6
1.71	140.044	2
1.77	176.068	3
2.23	205.082	9
2.29	170.057	4
2.74	140.033	4
2.85	193.071	6
2.90	197.055	6
3.72	194.054	6
4.42	155.057	6
7.38	178.062	4
7.49	139.062	2
7.80	175.074	2
7.90	179.080	10
8.15	175.074	1
8.34	220.095	2
8.47	202.084	2
1.25	174.973	-
1.46	226.952	-
7.06	174.973	-
10.12	171.100	-
11.80	239.090	-
11.96	134.072	-
12.06	185.115	-
13.79	229.051	-
15.13	227.126	-
15.78	233.106	-
16.74	245.079	-
16.95	348.991	-

Table S3. Picked peaks from the HPLC-MS data showing the intensities in each experimental run for H experiments. Retention times are in minutes and peak heights in counts. Isobaric peaks are not shown. **Part 1.**

rt	*Max m/z*	*m/z in D*	*PCA #*	MU.X1.D1	MU.X1.D1	MU.X1.D1	MU.X1.D2	MU.X1.D2	MU.X1.D2	MU.X1.D3	MU.X1.D3	MU.X1.D3	MU.X2.D1	MU.X2.D1	MU.X2.D1	MU.X2.D2	MU.X2.D2	MU.X2.D2	MU.X2.D3	MU.X2.D3	MU.X2.D3
7.38	178.0615	182.0866	1	28861	30760	31888	31862	30122	34631	42776	42640	44789	65258	66819	72079	74509	80194	74546	58004	67381	61657
2.29	170.0565	174.0816	2	11417	10099	10368	21904	25003	25440	41033	41646	40433	108226	118818	116922	46689	48475	44956	49369	47490	47837
1.89	134.0329	152.1458	3	235	301	253	312	242	271	6540	6285	5212	484	443	322	260	331	242	9113	9300	8184
1.60	148.061	155.1049	4	1310	1582	1724	3624	3721	3827	780	887	936	2246	2605	2256	7586	7899	8035	1853	1707	1826
3.72	194.0541	200.0917	5	49427	51598	48822	39791	40957	42968	12042	12198	12972	36472	35206	35396	35710	37493	35807	14084	14771	14247
4.42	155.0567	161.0943	6	32435	38120	38465	15537	18829	17936	39492	41610	40192	56426	63091	62890	37414	44704	41058	40801	42113	42172
7.80	175.0737	177.0862	7	15484	15393	15266	10692	10533	10504	60230	61907	62109	33627	33685	30835	26091	26818	27586	29671	28175	28357
2.85	193.071	199.1086	8	2921	3246	3529	23026	23314	23343	396	403	365	1406	1186	1318	3379	3568	3631	436	400	392
8.06	154.0734	167.1549	9	29828	30696	28782	9729	9010	9479	5935	6168	6170	15863	14954	15250	13095	11775	13051	7990	9109	9411
2.90	197.0545	203.0921	10	34776	33763	34608	29095	29266	28088	32285	34716	32746	69362	65953	68133	45481	48479	49160	35037	34428	35325
8.47	202.0842	204.0967	11	1007	945	993	981	885	922	19326	20082	20687	100721	102622	102694	3835	4469	4191	3005	3067	2792
8.34	220.0948	222.1073	12	343	406	406	417	492	381	41175	41556	41367	65752	72875	71200	2211	2352	2409	7036	6191	6986
1.71	140.0437	142.0562	13	5006	4863	4780	3184	2814	3157	3239	3046	3348	2833	3376	3391	3539	3445	3812	3229	3946	3584
7.49	139.0621	141.0746	14	949	934	860	2424	2382	2365	6589	7316	7743	22809	20642	22871	4812	5714	5730	5759	6349	6151
8.15	175.0738	176.0801	15	489	490	524	704	775	648	32444	32270	31730	7594	7358	6525	9547	10798	9604	28625	27543	28325
1.59	102.0562	109.1001	16	1197	1134	1496	6987	6746	7008	1796	1880	1882	1625	1468	1701	10298	9931	9798	6117	5919	5599
1.77	189.0188	212.163	17	5209	5021	4854	1332	1346	1266	946	940	967	1164	1002	972	458	408	476	521	588	569
4.59	154.0478	161.0917	18	32435	38120	38465	15537	18829	17936	39492	41610	40192	56426	63091	62890	37414	44704	41058	40801	42113	42172
1.53	154.0603	158.0854	19	7278	7382	7606	21627	19352	19742	4450	3870	4277	5132	4525	4277	3822	4131	3907	3503	3865	3702
1.66	213.0504	219.088	20	1985	1806	2117	2870	3037	2987	2465	2754	2440	5529	5365	5568	11526	12198	11505	4170	4364	4505
2.23	205.0821	214.1385	21	5910	5174	5321	14742	14804	14563	10175	9505	8838	15158	14847	15650	36241	36766	35391	24332	23418	23746
2.74	140.0326	144.0577	22	14232	14974	15942	2830	3003	3289	3025	3465	3420	7232	6929	6355	5936	6639	6837	4663	4229	4919
8.48	193.0349	206.1164	23	2889	3090	3305	7106	8378	7977	19884	22064	22443	14507	15404	15545	4778	4957	5810	6672	6955	7292
1.77	176.0676	179.0864	24	3506	2744	3527	2065	1856	1909	2676	2784	2571	5017	4781	5302	4457	4481	4617	3201	3130	3262
7.90	179.0797	189.1424	25	27923	29940	30355	10335	11156	11944	6569	6557	7051	13974	14356	13796	13603	14460	14718	10495	10414	10510

Table S4. Picked peaks from the HPLC-MS data showing the intensities in each experimental run for D experiments. Retention times are in minutes and peak heights in counts. Isobaric peaks are not shown. **Part 2.**

rt	*Max m/z*	*m/z in D*	*PCA #*	MU.X1.H1	MU.X1.H1	MU.X1.H1	MU.X1.H2	MU.X1.H2	MU.X1.H2	MU.X1.H3	MU.X1.H3	MU.X1.H3	MU.X2.H1	MU.X2.H1	MU.X2.H1	MU.X2.H2	MU.X2.H2	MU.X2.H2	MU.X2.H3	MU.X2.H3	MU.X2.H3
7.38	178.0615	182.0866	1	52966	60307	61820	208567	227771	227844	99846	118462	113296	102341	110118	118513	133444	137537	148798	151612	168168	199020
2.29	170.0565	174.0816	2	11445	11257	12488	32342	32864	34720	7513	7691	8319	9236	9310	9314	12870	13642	14543	3779	4031	3472
1.89	134.0329	152.1458	3	12462	11060	11020	28286	25278	27278	18295	18267	19225	19891	19188	18508	17956	16533	18652	12533	12797	13828
1.60	148.061	155.1049	4	3278	3149	3064	3175	3438	3036	3130	3141	3321	6777	7388	6124	7050	7072	7038	951	900	906
3.72	194.0541	200.0917	5	15333	17065	16122	9926	10397	10337	8096	9300	8711	31704	31822	32566	12445	14321	14006	412	308	375
4.42	155.0567	161.0943	6	3354	2861	3474	6975	7099	7012	1775	1871	1668	3567	4064	4101	3617	3839	4076	1987	2153	2166
7.80	175.0737	177.0862	7	24305	22568	22506	45555	46037	47043	14855	15856	14935	9013	9282	9331	14344	14498	13031	1852	1882	1483
2.85	193.071	199.1086	8	9500	12475	11991	2716	2250	2458	3517	3471	3528	9628	10580	11566	2745	3205	3034	366	430	355
8.06	154.0734	167.1549	9	3411	3664	3566	549	542	714	1632	2052	2100	1159	1045	1096	2562	2573	2837	853	844	783
2.90	197.0545	203.0921	10	10606	12171	11368	28156	28673	28978	13387	14040	15370	11349	10415	10567	17280	18850	21081	11070	12516	11479
8.47	202.0842	204.0967	11	1576	1825	1834	97437	94923	97218	2333	2454	2194	1327	1455	1553	2982	3272	3220	870	856	763
8.34	220.0948	222.1073	12	3277	3431	3464	79003	79622	84008	5356	6329	6403	1643	1479	1634	2582	2367	2425	305	290	365
1.71	140.0437	142.0562	13	2227	2346	2269	3349	3248	3196	4007	4039	4167	4168	4026	3780	4164	3986	4024	3095	3654	3135
7.49	139.0621	141.0746	14	4879	4291	5086	9095	9723	9669	2508	2777	2448	2024	2198	2140	4703	5445	6186	2773	3286	2858
8.15	175.0738	176.0801	15	2058	2603	2612	19538	19979	20038	1560	1628	2333	987	939	1234	454	2786	2198	158	240	261
1.59	102.0562	109.1001	16	2841	2809	2745	4946	4709	4675	4072	4303	4690	8107	7172	7770	6047	5570	6066	393	456	484
1.77	189.0188	212.163	17	1079	1023	1171	1261	1338	1339	2434	2423	2588	2738	2753	2275	2055	1862	1565	1679	1562	1516
4.59	154.0478	161.0917	18	2658	2743	3206	5028	4922	5350	4094	4357	4093	3299	4042	3787	5244	5244	5522	1463	1625	1608
1.53	154.0603	158.0854	19	5609	5891	5413	1739	1766	2021	5931	7384	6493	12098	11406	11508	3346	3315	3542	174	129	137
1.66	213.0504	219.088	20	1642	1491	1707	5185	5092	5308	1914	2318	2442	7304	6137	6711	2221	2760	2601	211	211	246
2.23	205.0821	214.1385	21	3030	3547	3275	7182	7571	6817	4678	4713	4737	6422	6454	6812	6595	6484	6204	823	934	1030
2.74	140.0326	144.0577	22	1250	1397	1109	1640	1880	1975	274	354	255	2347	2392	2226	2990	2822	2996	1801	1513	1734
8.48	193.0349	206.1164	23	1814	1705	1856	1189	1348	1364	4411	4635	4750	5669	6308	6466	10726	11003	11864	3516	3365	3271
1.77	176.0676	179.0864	24	1167	1251	1045	2767	2985	2703	894	1144	1152	1227	1382	1486	2177	2247	2480	1484	1624	1369
7.90	179.0797	189.1424	25	2753	3043	3496	3784	4518	4595	1813	2142	1773	1573	1500	1446	3975	4246	3842	3733	3769	4622

Table S5. Tentative assignment of chemical formulae for peaks found in both H and D experiments with a matching *rt* and integer number of [D-H] masses difference (see Table S2). Formula fitting and matching was performed using R and MS Excel. Pairs of formulae corresponding to the matched masses were selected so as to have the lowest total PPM error. All formulae are fitted to the *m/z* value and assumed to represent $[M+H]^+$ ions.

1.53	154.0603	4883	$C_4H_6N_6O$	0.06	1.51	158.0842	7358	$C_4D_4H_2N_6O$	7.70	4
1.59	102.0562	4325	$C_4H_8NO_2$	-6.82	1.60	109.0994	4588	$C_4D_7HNO_2$	0.39	7
1.60	148.061	4052	$C_5H_{10}NO_4$	-0.12	1.60	155.1053	3022	$C_5D_7H_3NO_4$	-2.44	7
1.66	213.0504	3083	$C_6H_7N_5O_4$	-2.80	1.64	219.0868	4844	$C_6D_6HN_5O_4$	3.04	6
1.71	140.0437	3493	$C_2H_8N_2O_5$	-2.70	1.72	142.0564	3588	$C_2D_2H_6N_2O_5$	-3.69	2
1.77	176.0676	1699	$C_5H_{10}N_3O_4$	-2.66	1.78	179.0872	3438	$C_5D_3H_7N_3O_4$	-6.91	3
2.23	205.0821	4850	$C_7H_{13}N_2O_5$	1.69	2.12	214.1387	17477	$C_7D_9H_4N_2O_5$	1.12	9
2.29	170.0565	13269	$C_6H_8N_3O_3$	0.39	2.25	174.0815	47563	$C_6D_4H_4N_3O_3$	1.00	4
2.74	140.0326	1720	$C_6H_6NO_3$	15.48	2.70	144.0574	6551	$C_6D_4H_2NO_3$	17.19	4
2.85	193.071	5212	$C_6H_7N_7O$	1.08	2.74	199.1078	5348	$C_6D_6HN_7O$	5.37	6
2.90	197.0545	15964	$C_5H_{11}NO_7$	-4.81	2.84	203.0914	41150	$C_5D_6H_5NO_7$	-0.92	6
3.72	194.0541	13514	$C_5H_{10}N_2O_6$	-1.10	3.64	200.0917	31665	$C_5D_6H_4N_2O_6$	-0.76	6
4.42	155.0567	3648	$C_5H_7N_4O_2$	1.29	4.46	161.0918	39627	$C_5D_6HN_4O_2$	17.15	6
7.38	178.0615	135579	$C_8H_8N_3O_2$	0.85	7.30	182.0874	52154	$C_8D_4H_4N_3O_2$	-3.52	4
7.49	139.0621	4561	$C_5H_7N_4O$	-0.82	7.48	141.0753	7356	$C_5D_2H_5N_4O$	-5.39	2
7.80	175.0737	18243	$C_9H_9N_3O$	4.92	7.76	177.0856	29276	$C_9D_2H_7N_3O$	8.56	2
7.90	179.0797	3146	$C_6H_{13}NO_5$	-1.83	7.85	189.1435	14342	$C_6D_{10}H_3NO_5$	-7.18	10
8.15	175.0738	4534	$C_9H_9N_3O$	4.35	8.17	176.0794	13111	$C_9DH_8N_3O$	8.17	1
8.34	220.0948	15777	$C_{10}H_{12}N_4O_2$	5.57	8.27	222.1073	20198	$C_{10}D_2H_{10}N_4O_2$	5.76	2
8.47	202.0842	17672	$C_7H_{12}N_3O_4$	-7.02	8.52	204.0965	21846	$C_7D_2H_{10}N_3O_4$	-5.71	2

Table S6. Tentative assignment of chemical formulae for peaks found only in H experiments. Formula fitting and matching was performed using R and MS Excel. Formulae were picked for lowest PPM error. All formulae are fitted to the *m/z* value and assumed to represent $[M+H]^+$ ions.

rt /min	max. m/z	Intensity	Fitted Formula	ppm error
1.53	255.9793	1575	$C_4H_4N_2O_{11}$	8.63
1.59	169.0591	2516	$C_4H_{11}NO_6$	-2.74
1.71	118.0627	2243	$C_3H_8N_3O_2$	-8.88
1.75	367.0031	1381	$C_{11}H_5N_5O_{10}$	1.48
1.77	364.9971	1482	$C_7H_5N_6O_{12}$	-1.52
1.77	189.0188	1815	$C_8H_3N_3O_3$	-7.19
1.82	205.0827	1343	$C_7H_{13}N_2O_5$	-1.24
1.82	248.0895	1200	$C_9H_{10}N_7O_2$	0.39
1.89	134.0329	17837	$C_3H_6N_2O_4$	-1.07
1.94	246.0742	2325	$C_9H_8N_7O_2$	-1.03
1.99	142.0629	1319	$C_5H_8N_3O_2$	-8.79
2.23	148.0486	7943	$C_4H_8N_2O_4$	-1.30
2.37	171.0414	12864	$C_6H_7N_2O_4$	-4.78
3.60	139.0621	3599	$C_5H_7N_4O$	-0.82
3.63	172.0358	5175	$C_5H_6N_3O_4$	0.18
3.88	205.0336	3675	$C_5H_7N_3O_6$	-0.56
3.88	162.0642	3083	$C_5H_{10}N_2O_4$	-0.88
4.54	195.0881	4547	$C_8H_{11}N_4O_2$	0.52
4.59	154.0478	3794	$C_3H_4N_7O$	-0.44
5.32	150.0641	9022	$C_4H_{10}N_2O_4$	-0.29
5.38	177.0388	4384	$C_4H_7N_3O_5$	-1.30
5.68	162.0645	619	$C_5H_{10}N_2O_4$	-2.73
6.04	171.0519	2957	$C_5H_7N_4O_3$	-0.50
7.03	208.0698	1502	$C_6H_{12}N_2O_6$	-1.27
7.49	160.0238	1018	$C_3H_4N_4O_4$	-3.41
7.51	161.0445	5431	$C_4H_7N_3O_4$	-5.24
7.68	169.0838	3456	$C_5H_9N_6O$	-0.09
7.80	215.0425	1971	$C_7H_3N_8O$	2.24
7.84	203.0680	2683	$C_8H_7N_6O$	0.66
7.91	273.0849	2362	$C_{11}H_{15}NO_7$	-0.18
7.91	198.0636	7073	$C_8H_{10}N_2O_4$	2.31
7.92	153.0785	3984	$C_8H_{11}NO_2$	3.13
8.00	191.0549	1848	$C_6H_5N_7O$	3.44
8.06	154.0734	1777	$C_5H_8N_5O$	-3.34
8.10	219.0614	8648	$C_6H_5N_9O$	1.40
8.20	319.0907	4516	$C_{12}H_{17}NO_9$	-1.16
8.48	193.0349	4737	$C_5H_3N_7O_2$	-0.40
8.55	192.0781	5863	$C_9H_{10}N_3O_2$	-4.16
8.77	263.1006	4970	$C_{10}H_{17}NO_7$	-0.37

Table S7. Tentative assignment of chemical formulae for peaks found only in D experiments. Formula fitting and matching was performed using R and MS Excel. Formulae were picked for lowest PPM error. All formulae are fitted to the *m/z* value and assumed to represent $[M+H]^+$ ions.

rt /min	max. m/z	Intensity	Fitted Formula	ppm error
1.60	154.1217	3638	$C_3D_6H_4N_5O_2$	-3.82
1.70	230.0689	3128	$C_5D_4H_6N_2O_8$	-0.33
1.70	213.0998	2026	$C_{13}D_2H_9N_2O$	-0.51
1.77	118.0923	2074	$C_4D_4H_4N_3O$	-3.85
1.79	212.1819	1558	$C_8D_{10}H_6N_3O_3$	0.17
1.80	278.1210	2412	$C_8DH_{10}N_{11}O$	0.29
1.83	177.0826	8385	$C_9D_5HN_3O$	-0.72
1.83	136.0462	8452	$C_6D_3N_3O$	1.78
1.84	171.1002	19884	$C_3D_6H_3N_4O_4$	-0.93
1.88	114.0636	9227	$C_4D_2H_4N_3O$	0.36
1.91	170.1158	47524	$C_5D_7H_4N_2O_4$	0.12
1.93	171.1554	7806	$C_6D_{10}H_3N_2O_3$	-0.08
1.96	189.1260	4510	$C_5D_5H_5N_7O$	0.36
1.96	152.1610	2685	$C_6D_{10}H_2N_3O$	-1.27
2.12	236.1209	16692	$C_{13}D_5H_8NO_3$	0.12
2.22	131.0758	13895	$C_5D_4H_3N_2O_2$	0.46
2.29	241.1074	2068	$C_4D_4H_7N_7O_5$	-0.52
2.34	178.0656	4803	$C_4D_3H_4N_4O_4$	-0.22
3.03	187.1506	21985	$C_4D_9H_3N_5O_3$	-0.50
3.16	154.0862	2163	$C_2D_5H_2N_5O_3$	0.48
3.34	192.0918	12729	$C_9D_6N_4O$	0.12
3.44	170.1716	10256	$C_4D_9H_4N_6O$	-0.14
3.72	250.0788	2596	$C_7DH_6N_9O_2$	-1.01
3.73	147.1041	5981	$C_6D_6H_3N_2O_2$	-0.24
3.73	229.2070	1942	$C_8D_{11}H_7N_4O_3$	-0.27
4.34	135.0817	10112	$C_5D_5H_3NO_3$	0.76
4.99	157.1088	25830	$C_2D_6H_3N_5O_3$	-3.81
7.51	117.1157	4199	$C_2D_6H_5N_2O_3$	-9.14
7.54	163.1335	4068	$C_7D_7H_5N_2O_2$	1.94
7.80	177.1200	6914	$C_3D_8H_3N_3O_5$	0.49
7.85	167.1604	13683	$C_7D_{10}H_3N_2O_2$	0.43
7.92	188.1361	7495	$C_4D_8H_4N_4O_4$	-0.16
8.17	151.1099	12420	$C_6D_7H_3NO_3$	0.71
8.18	159.1266	5478	$C_4D_5H_9N_2O_4$	0.89
8.27	201.0510	4066	$C_8DH_3N_6O$	-0.32
8.48	196.0528	3075	$C_2D_2H_4N_6O_5$	-1.42
8.53	213.1299	4530	$C_3D_8HN_8O_3$	0.36
8.55	158.1200	6183	$C_7D_6H_4N_3O$	0.31
8.62	183.1886	7287	$C_8D_{12}H_3N_2O_2$	0.42
8.63	206.1199	9948	$C_2D_9N_6O_5$	0.17
9.01	167.1389	3290	$C_3D_7H_3N_7O$	-1.66
9.61	148.1436	4853	$C_7D_9H_2N_2O$	0.21
10.75	165.1780	7439	$C_8D_{12}HN_2O$	0.68

Table S8. Tentative assignment of chemical formulae for isobaric peaks found in both H and D experiments. Formula fitting and matching was performed using R and MS Excel. Formulae were picked for lowest PPM error. All formulae are fitted to the *m/z* value and assumed to represent $[M+H]^+$ ions.

rt /min	max. m/z	Intensity	Fitted Formula	ppm error
7.56	198.0629	25382	$C_6H_8N_5O_3$	-0.94
8.10	197.0786	10636	$C_6H_9N_6O_2$	0.50
8.47	174.0711	1659	$C_3H_8N_7O_2$	16.36
8.55	217.1055	4611	$C_6H_{13}N_6O_3$	-2.70
10.12	171.0996	8253	$C_5H_{11}N_6O$	-0.97
11.80	239.0897	33428	$C_8H_{11}N_6O_3$	-1.83
11.96	134.0717	7332	$C_4H_{10}N_2O_3$	-19.08
12.06	185.1154	10489	$C_6H_{13}N_6O$	-1.71
13.79	229.0511	6513	$C_{16}H_7NO$	7.26
15.13	227.1257	27708	$C_8H_{15}N_6O_2$	-0.23
15.78	233.1055	3379	$C_{13}H_{15}NO_3$	-1.32
16.74	245.0788	4044	$C_{10}H_9N_6O_2$	-0.41
16.95	348.9911	14720	$C_{10}H_7NO_{13}$	1.83
16.95	350.9881	14046	$C_{16}H_3N_2O_8$	2.39
